# The new platinum(IV) derivative LA-12 shows stronger inhibitory effect on Hsp90 function compared to cisplatin

**DOI:** 10.1186/1476-4598-9-147

**Published:** 2010-06-15

**Authors:** Veronika Kvardova, Roman Hrstka, Dawid Walerych, Petr Muller, Eva Matoulkova, Veronika Hruskova, Dagmar Stelclova, Petr Sova, Borivoj Vojtesek

**Affiliations:** 1 Department of Oncological and Experimental Pathology, Masaryk Memorial Cancer Institute, Zluty kopec 7, 656 53 Brno, Czech Republic; 2 Department of Molecular Biology, International Institute of Molecular and Cell Biology in Warsaw, 4 Ks. Trojdena Street, 02-109 Warsaw, Poland; 3 Research and Development, PLIVA-Lachema a.s., Karásek 1, 621 33 Brno, Czech Republic

## Abstract

**Background:**

Cisplatin and its derivatives are commonly used anti-cancer drugs. However, cisplatin has clinical limitations including serious side effects and frequent emergence of intrinsic or acquired resistance. Thus, the novel platinum(IV) complex LA-12 represents a promising treatment modality, which shows increased intracellular penetration resulting in improved cytotoxicity in various cancer cell lines, including cisplatin resistant cells.

**Results:**

LA-12 disrupts cellular proliferation regardless of the p53 status in the cells, however the potency of the drug is greatly enhanced by the presence of a functional p53, indicating several mechanisms of action. Similarly to cisplatin, an interaction of LA-12 with molecular chaperone Hsp90 was proposed. Binding of LA-12 to Hsp90 was demonstrated by Hsp90 immunoprecipitation followed by platinum measurement using atomic absorption spectrometry (AAS). An inhibitory effect of LA-12 on Hsp90 chaperoning function was shown by decrease of Hsp90-assisted wild-type p53 binding to p21^WAF1 ^promoter sequence *in vitro *and by accelerated ubiqutination and degradation of primarily unfolded mutant p53 proteins in cells exposed to LA-12.

**Conclusions:**

To generalize our findings, LA-12 induced degradation of other Hsp90 client proteins such as Cyclin D1 and estrogen receptor was shown and proved as more efficient in comparison with cisplatin. This newly characterised molecular mechanism of action opens opportunities to design new cancer treatment strategy profitable from unique LA-12 properties, which combine DNA damaging and Hsp90 inhibitory effects.

## Background

Cisplatin [cis-diamminedichloroplatinum(II)] is a platinum-based anticancer drug commonly used for treatment of different types of cancer, including ovarian, testicular, head and neck or lung carcinomas [1]. Although cisplatin exerts significant anti-tumour activity, cisplatin-based therapies cause numerous side effects, such as nephrotoxicity, neurotoxicity and nausea. The intrinsic as well as acquired resistances to cisplatin-based therapy also represent substantial complications for the treatment of tumours.

For this reason, vast efforts were committed to develop novel platinum-based complexes to overcome platinum resistance, to reduce cisplatin side effects and to introduce novel mechanisms of anti-cancer action. Two derivatives, carboplatin (cis-diammine-(1,1-cyclobutanedicarboxylato)platinum(II)) and oxaliplatin (trans-R,R.cyclohexane-1,2-diammine)oxalatoplatinum(II)), have been introduced [2]. Taken together, these three drugs represent all of the available platinum drugs approved by the Food and Drug Administration for clinical use [3]. In addition to substantial side effects, these three drugs also require intravenous administration, therefore a new generation of platinum drugs represented by satraplatin (JM216) [(*OC*-6-43)-bis(acetato)amminedichloro(cyclohexylamine)platinum(IV)] has been generated. JM216 represents the first orally administered platinum compound which has been evaluated in clinical trials [2,4,5].

LA-12, [(*OC*-6-43)-bis(acetato)(1-adamantylamine)amminedichloroplatinum (IV)], is a hydrophobic platinum(IV) complex structurally similar to JM216, which contains 1-adamantylamine instead of cyclohexylamine non-leaving ligand [6] which provides this drug different properties. LA-12 has shown a higher cytotoxicity than JM216 when tested on a panel of 14 cancer cell lines of various origin and different cisplatin sensitivity [6,7] and no cross-resistance with cisplatin [6,8]. LA-12 has also displayed (i) higher antitumor activity in comparison with cisplatin and JM216, (ii) favourable pharmacokinetics and (iii) relatively low acute toxicity in a panel of pre-clinical *in vivo *studies [9-11].

The cytotoxic mode of action of cisplatin is mediated by its interaction with DNA to form DNA adducts, primarily intra-strand crosslink adducts, which activate several signal transduction pathways, including those involving ATR, p53, p73, and MAPK, and culminate in the activation of apoptosis [12]. JM216 has a mechanism of action similar to that of cisplatin, inducing the formation of DNA adducts and inter- and intra-strand crosslinks and resulting in G2 arrest and induction of apoptosis [13]. The principle mechanisms of LA-12 effects are not fully understood to date, although there is some evidence that exposure to LA-12 can disrupt cell proliferation and induce apoptosis more potently than cisplatin in both p53 dependent and independent manners [14,15]. Interestingly, LA-12 induces unique changes in the profile of gene expression compared to cisplatin, indicating a distinct mode of action resulting in the differential activation of both p53-dependent and p53-independent gene targets [16].

Besides the well known cisplatin DNA-mediated effect, the other mechanism of cisplatin action was described demonstrating that cisplatin may also disrupt the function of some proteins, including heat shock protein 90 (Hsp90) [17]. According to this study, cisplatin associates with the molecular chaperone Hsp90 and reduces its' chaperone activity. For example, this can result in the degradation of steroid receptors [18] and/or inhibition of interaction between Hsp90 and inositol hexakisphosphate kinase-2 (IP6K2), which disrupts the inhibitory effect of Hsp90 on IP6K2, leading to increased diphosphoinositol pentakisphosphate (IP7) formation and sensitisation of cancer cells to apoptosis [19,20].

Since it is supposed that LA-12 undergoes metabolic changes and serves actually as a pro-drug for chemotherapeutically active platinum analogues, it is possible that these active metabolites can interact with proteins analogous to cisplatin, resulting in stronger cytotoxic or cytostatic effects. According to this hypothesis we have analyzed whether LA-12 can bind to Hsp90 resulting in inhibition of its protective chaperoning function such as folding, stabilization, activation and assembly of its "client" proteins.

## Results

### Time and dose dependent accumulation of LA-12 in cancer cells in comparison with cisplatin

LA-12 and cisplatin uptake into cells was determined by AAS platinum amount measurement in lysates of cells treated with different doses of these drugs for different periods of time. We observed rapid and high initial accumulation of platinum after 0.5 hour incubation with LA-12 in all concentrations used, rising up to 4 hours of incubation. In subsequent time points, platinum level remained stable. The platinum amount in cells was also dependent on the concentration used.

**Figure 1:**
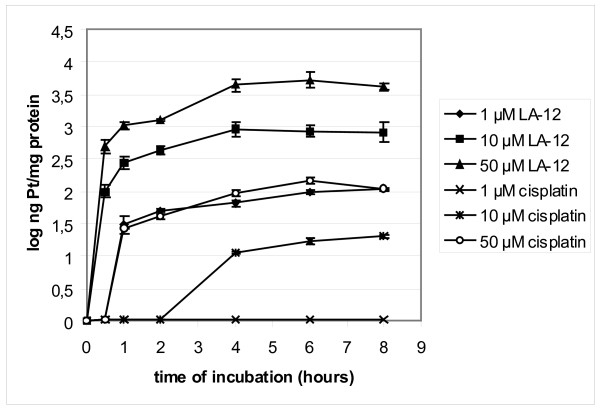
**Platinum accumulation in H1299 cells**. H1299 cells were treated with varying concentrations of LA-12 and cisplatin for indicated time intervals. Platinum levels in cell lysates were analysed by AAS. Results are mean ± SD of triplicate experiments.

In contrast, there was no platinum detected in response to 1 μM cisplatin treatment. Compared to LA-12 exposure, platinum levels were detected only when 10 μM and 50 μM cisplatin doses were administered. In response to LA-12, platinum levels were 30 to 50 times higher depending on concentration used and time interval (Figure 1). These results demonstrate that cellular uptake of LA-12 is faster and more effective compared to cisplatin.

### LA-12 specifically interacts with Hsp90

**Figure 2:**
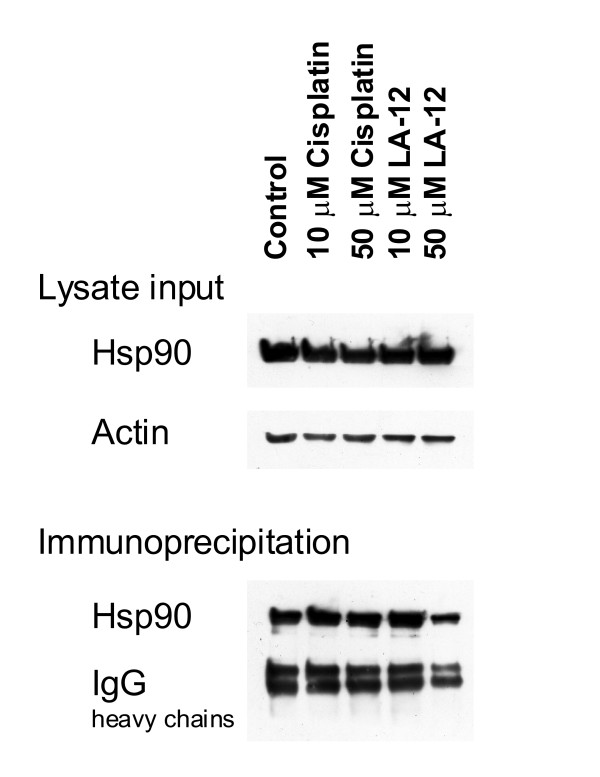
**Detection of Hsp90 protein**. The levels of Hsp90 protein in H1299 whole cell lysates and after immunoprecipitation of Hsp90 in LA-12 and cisplatin treated cells were analysed by western blotting. EEV1-2.1 mouse monoclonal antibody was used for detection of Hsp90 protein.

To assess the anticipated interaction of Hsp90 with LA-12, we measured in triplicate the amount of platinum bound to Hsp90 by AAS after immunoprecipitation of Hsp90 from H1299 cells treated with 10 and 50 μM both LA-12 and cisplatin. We detected significant amounts of platinum after LA-12 treatment in contrast to cisplatin treatment, where only after using 50 μM concentration was a limited amount of platinum detected (about detection limit of AAS) (Table 1). The presence of Hsp90 in the samples after immunoprecipitation was confirmed using western blot (Figure 2). These data show that LA-12 binds to Hsp90 more effectively than cisplatin *in vivo*, probably due to the higher intracellular levels of LA-12.

**Table 1:** Analysis of interaction of Hsp90 with LA-12

Sample	Applied	Result (± SD)	Result
	compound	mg Pt/ml	ng Pt/mg protein
D-MEM	negative	-	
10 μM cisplatin	10 μM cisplatin	2.09 (0.05)	
50 μM cisplatin	50 μM cisplatin	9.93 (0.21)	
10 μM LA-12	10 μM LA-12	1.96 (0.01)	
50 μM LA-12	50 μM LA-12	9.14 (0.26)	

L1	negative	-	
L2	10 μM cisplatin	0.040 (0.0004)	20
L3	50 μM cisplatin	0.290 (0.005)	145
L4	10 μM LA-12	1.98 (0.02)	990
L5	50 μM LA-12	11.6 (0.26)	5500

P1	negative	-	
P2	10 μM cisplatin	-	
P3	50 μM cisplatin	< 0.02	
P4	10 μM LA-12	0.034 (0.004)	
P5	50 μM LA-12	0.222 (0.01)	

NC1	50 μM cisplatin	-	
NC2	50 μM LA-12	-	
AC1	50 μM cisplatin	-	
AC2	50 μM LA-12	-	

Platinum levels were determined by AAS in whole cell lysates (L1-5) of H1299 control cells and cells treated with 10 μM and 50 μM LA-12 or cisplatin for 9 hours. The protein concentration of cell lysates was 2 mg/ml. The amounts of platinum bound to Hsp90 was measured after immunoprecipitation of Hsp90 from H1299 control cells and cells treated with 10 μM and 50 μM LA-12 or cisplatin for 9 hours (P1-5). Immunoprecipitations without antibody (NC1-2) and with DO-1 antibody (AC1-2) in cells treated with 50 μM LA-12 and cisplatin were performed as negative control for crossreactivity of platinum compounds with other reaction components. Control AAS platinum measurements were perfomed in D-MEM cell medium containing 10 μM and 50 μM LA-12 or cisplatin. Results are mean ± SD of triplicate experiments.

### Inhibition of Hsp90 assisted p53 binding to promoter sequence of p21^WAF1 ^by LA-12

**Figure 3:**
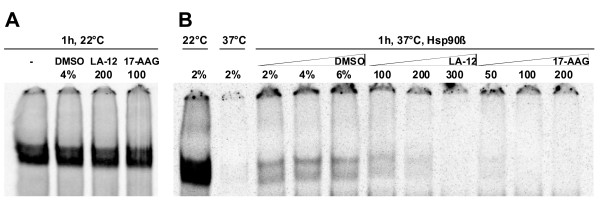
**The Hsp90-dependent binding of wild-type p53 protein to p21^WAF1 ^gene promoter sequence is inhibited by LA-12 and 17-AAG**. A) Wild-type p53 binding (0.125 μM tetramer) to the promoter sequence at 22°C (the main band) is not influenced by 4% DMSO (final concentration in the reaction) or LA-12 and 17-AAG dissolved in the corresponding DMSO amount. B) Hsp90β (3 μM dimer) with 5 mM ATP (final concentration in all reactions) rescues the p53 activity from inactivation at 37°C, in the presence of an increasing DMSO concentration, while LA-12 and 17-AAG inhibit Hsp90β. The LA-12 stock solution is 2× more concentrated than 17-AAG to compensate for the lower LA-12 activity. Thus both inhibitors are in corresponding DMSO amounts: 100, 200, 300 μM LA-12 and similarly 50, 100, 200 μM 17-AAG introduce 2%, 4%, 6% DMSO concentration to the reaction, respectively.

The influence of LA-12 on the ability of wild-type p53 to specifically bind to its target sequence derived from the p21^WAF1 ^gene promoter was tested using an electrophoretic mobility shift assay (EMSA). It is known that human wild-type p53 binding activity is facilitated by molecular chaperone Hsp90 under physiological conditions and this process is inhibited by geldanamycin [21]. Thus in 22°C wild-type p53 tetramers can bind to the p21^WAF1 ^promoter independently of Hsp90 status. Under physiological conditions (37°C) in a control reaction without Hsp90, human wild-type p53 loses its ability to bind to the p21^WAF1 ^promoter; however in presence of human Hsp90β and ATP, wild-type p53 can develop transcriptionally active tetramers that bind to the p21^WAF1 ^promoter sequence. In consequence, when Hsp90β is inhibited by 17-AAG, the affinity of wild-type p53 to p21^WAF1 ^promoter is disrupted. Similarly, in a case of LA-12 we also observed the disruption of Hsp90β activity leading to inhibition of wild-type p53 DNA binding affinity (Figure 3). Cisplatin could not be effectively compared to LA-12 and 17-AAG in this assay, as it directly disrupts the p53 binding to promoter sequence (not shown).

### LA-12 inhibits Hsp90 assisted folding and initiates degradation of unfolded proteins

**Figure 4:**
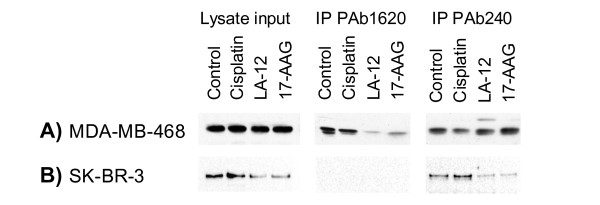
**Analysis of p53 mutant protein conformation**. p53 mutant protein conformation was analysed in A) MDA-MB-468 cells containing mostly folded p53 R273H mutant and B) SK-BR-3 cells containing only unfolded p53 R175H mutant. Cells were treated for 9 hours with 10 μM cisplatin, 10 μM LA-12 and 4 μM 17-AAG. Conformational changes of p53 were determined using immunoprecipitation with p53 conformation-specific mouse monoclonal antibodies PAb1620 (detects folded conformation) and PAb240 (detects unfolded conformation). CM-1 rabbit polyclonal antibody was used for detection of p53 protein.

Hsp90 is important for folding and stabilization/degradation of client proteins. In our previous work we demonstrated that particular p53 mutations show different ratios between native wild-type p53 conformations (recognized by PAb1620 antibody) and denatured conformations (recognized by PAb240 antibody) and highlighted an important role of Hsp90 to maintain the mutant p53 protein conformation and stability [22]. In the case of partially folded p53 mutants, such as R273H naturally expressed in MDA-MB-468 cells, the native conformation is maintained by Hsp90. Thus, inhibition of Hsp90 results in a decrease of R273H mutant p53 folded conformation and an increase of denatured conformation, although the overall protein levels are not changed. We found that LA-12 is also able to cause the same changes in R273H folded p53 mutant conformation without appreciably affecting the level of total p53 protein (Figure 4A). In the case of predominantly unfolded mutant p53 proteins, such as R175H naturally expressed in SK-BR-3 cells, inhibition of Hsp90 function results in their degradation, and the identical effect is seen after treatment with LA-12 (Figure 4B).

### Mechanism of mutant p53 protein degradation in response to LA-12

**Figure 5:**
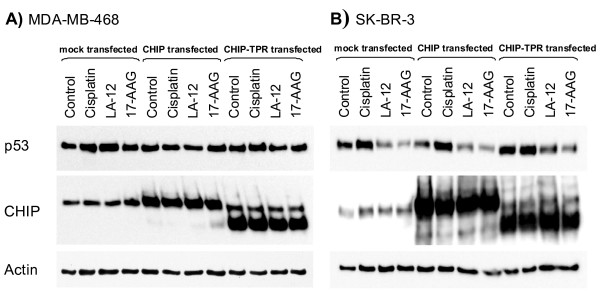
**Analysis of p53 mutant protein degradation**. p53 mutant protein levels were analysed in A) MDA-MB-468 cells containing mostly folded p53 R273H mutant and B) SK-BR-3 cells containing unfolded p53 R175H mutant transfected with CHIP or CHIP-TPR protein by western blotting. Cells were treated for 9 hours with 10 μM cisplatin, 10 μM LA-12 and 4 μM 17-AAG. CM-1 rabbit polyclonal antibody and CHIP-11.1 mouse monoclonal antibody were used for detection of p53 and CHIP proteins. Actin was used as loading control.

In our previous work we have shown that naturally unfolded mutants, such as p53 R175H in SK-BR-3 cells, form stable complexes with chaperones and that Hsp90 activity is crucial to prevent their degradation [22]. If the Hsp90 activity is blocked by 17-AAG, the denatured mutants develop complex with Hsp70 and CHIP proteins, where ubiquitin ligase CHIP is responsible for ubiquitination of unfolded p53 and ubiqutinated p53 protein is targeted for degradation in the proteasome [22,23]. To investigate whether LA-12 also initiates unfolded p53 mutant degradation via activation of CHIP ubiquitin ligase, we compared mutant p53 levels in cells transiently transfected by CHIP and a dominant-negative CHIP-TPR truncated form, which contains the TPR domain (responsible for interactions with chaperones) but lacks the ubiquitination domain. In MDA-MB-468 cells with a partially unfolded p53 mutant that is independent of Hsp90 activity, there was no effect of 17-AAG or LA-12 in the presence or absence of CHIP or CHIP-TPR (Figure 5A). In mock-transfected SK-BR-3 cells, we observed a decrease of mutant p53 in response to both LA-12 and 17-AAG, as well as in cells transfected by CHIP. Importantly, transfection by CHIP-TPR decreased the rate of degradation of the unfolded mutant p53, indicating the role of CHIP ubiquitn ligase in degradation of R175H p53 mutant caused by LA-12 (Figure 5B).

### LA-12 induces degradation of not only mutant p53 but also of other Hsp90 client proteins

**Figure 6:**
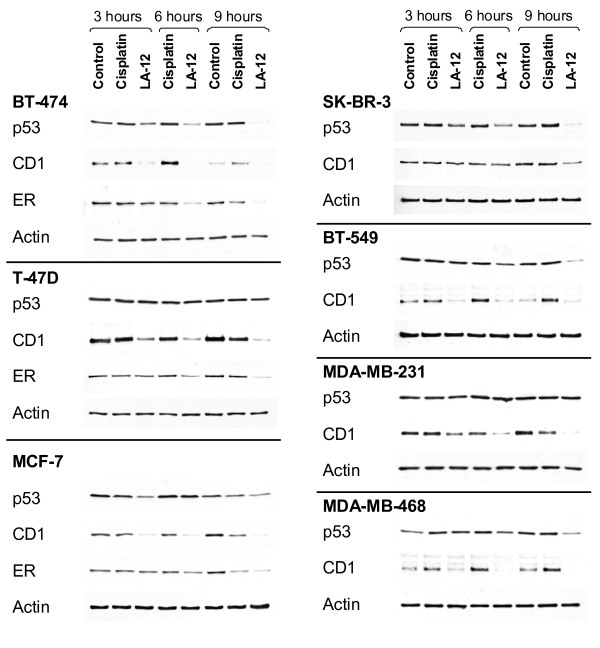
**Analysis of degradation of Hsp90 client proteins**. Protein levels of p53, cyclin D1 (CD1) and estrogen receptor (ER) were determined in cells treated for 3, 6 and 9 hours with 10 μM cisplatin and 10 μM LA-12 by western blotting. CM-1 rabbit polyclonal antibody, SP1 rabbit monoclonal antibody and CD1 mouse monoclonal antibody was used for detection of p53, ER and CD1 proteins, respectively. Actin was used as loading control.

Accordingly we found that LA-12 is responsible for massive decrease of p53 mutant proteins that adopt a predominantly denatured conformation (BT-474 and SK-BR-3 cells); in cells which carry both native and denatured conformations of mutant p53 (T-47D, BT-549 and MDA-MB-468), the decrease of p53 level was noticeable only after longer incubations (9 hours) with the drug. In MDA-MB-231 cells, where mutant p53 occurs predominantly in native conformation, no decrease of p53 level was observed even after prolonged incubation. Interestingly, apart from p53 protein, LA-12 also induces degradation of other Hsp90 client proteins, such as Cyclin D1 and Estrogen Receptor (Figure 6).

Importantly, the 10 μM drug dose used in our experiments is equal to 1.95 mg/l platinum, which corresponds to the plasma levels of platinum shown in previous *in vivo *studies [10,24]. The maximum platinum plasma concentration 1.54 and 6.25 mg/l in rats was observed within 1 hour after oral administration of 38.6 and 540 mg/kg LA-12, respectively [24]. Interestingly, maximum tolerated single oral dose of LA-12 in mouse model was 1000 mg/kg [10].

## Discussion

Platinum(IV) complexes represent a class of anticancer agents that are activated by reactions with reducing agents present in body liquids after their administration. Thus, the platinum(IV) complexes display potential advantages over their platinum(II) counterparts because of their greater stability and bioreductive activation, thereby allowing a greater proportion of the drug to arrive at the target intact. Thus, it is not surprising that the cellular uptake of LA-12 was faster and more effective than that of cisplatin (Figure 1).

Hsp90 is a molecular chaperone and is one of the most abundant proteins expressed in cells [25]. Molecular chaperone Hsp90 is a member of the heat shock protein family, which is up-regulated in response to stress and its' *in vivo *function is ATP-dependent [26]. Hsp90 exists as a homodimer, which contains three domains. The N-terminal domain contains an ATP-binding site that binds the natural products geldanamycin and radicicol. Hsp90 function depends upon the ability of the N-terminal domain to bind and hydrolyze ATP, which is regulated and coupled with the conformational changes of the Hsp90 dimer [27]. The middle domain exhibits high affinity for co-chaperones as well as client proteins [28]. The structure of the C-terminus of Hsp90 contains the MEEVD sequence, which is known to bind co-chaperones that contain multiple copies of the tetratricopeptide repeat (TPR), a 34 amino acid sequence [29]. Interestingly, the middle C-terminal part of Hsp90 has been implicated biochemically as the site of a putative second cryptic ATP-binding site on Hsp90 [26]. The contribution of this site to the overall regulation of chaperone function is not clear, but novobiocin has been reported to interact with this site and alter the conformation of the chaperone [30]. Importantly, cisplatin has also been found to bind to an Hsp90 site that overlaps the putative ATP/novobiocin binding site in this region, leading to inhibition of some Hsp90 activities [17,26]. The observed higher affinity of Hsp90 to LA-12 may be explained by very specific properties of C-terminal domain of Hsp90. This domain contains several solvent-exposed hydrophobic residues, which are responsible for binding of client protein such as unligated glucocorticoid receptor [31]. Consequently the hydrophobic properties of LA-12 could explain its preferential binding to Hsp90 in contrast to cisplatin.

The ability of LA-12 to inhibit Hsp90 function was shown using several approaches. (i) EMSA, based on the fact that under physiological conditions the chaperone activity of Hsp90 is important for the transcriptional activity of genotypically wild-type p53 [21] clearly demonstrated LA-12 as potent inhibitor of Hsp90 chaperoning function. (ii) Determination of p53 conformation in cells expressing various p53 mutants, which were exposed to LA-12, revealed that the unfolded mutant p53 proteins that do not rely on Hsp90 activity are less stable than folded mutant p53 proteins that require Hsp90 activity. These findings are in agreement with previous work showing that the half-life of p53 mutant positively correlates with their conformational stability [22].

It was shown that inhibition of Hsp90 results in ubiquitination and degradation of primarily unfolded p53 mutants. This MDM2-independent degradation of unfolded p53 mutant is mediated by chaperone interacting protein CHIP. Our results show that CHIP is responsible for LA-12 mediated degradation in the same way as 17-AAG, which supports our theories finding LA-12 as a specific Hsp90 inhibitor.

Accordingly to both higher cellular uptake of LA-12 and at least 10-fold higher presence of platinum bound to Hsp90 in cells exposed to LA-12 compared to cells treated by cisplatin (Table 1), a stronger suppressive effect of LA-12 on Hsp90 activity and function in relation to cisplatin could be expected. This statement was confirmed by the ability of LA-12 to influence the stability and degradation rate of other Hsp90 client proteins, such as estrogen receptor and cyclin D1 compared to cisplatin, which did not decrease the level of these proteins.

## Conclusions

In summary, our data indicate that cellular uptake of LA-12 is more effective compared to cisplatin resulting in more extensive binding of LA-12 to Hsp90 *in vivo*. This interaction is responsible for direct inhibition of Hsp90 chaperone function. These unique properties predict LA-12 as a novel promising anti-cancer therapeutic exploiting dual mode of action in inducing genotoxic damage and inhibiting Hsp90 activity.

## Methods

### Cell cultures

All human cancer cell lines were obtained from ATCC. Cell lines BT-474, MDA-MB-468, MDA-MB-231, T-47D, BT-549 and SK-BR-3 contain different p53 mutations (E285K, R273H, R280K, L194F, R249S and R175H, respectively), whereas the wild-type allele was lost in all these cell lines. MCF-7 cells contain wild-type p53, and H1299 cells are p53-null. Cells were periodically checked for morphology by microscope and regularly screened for mycoplasma using Hoechst staining.

Cells were cultured in Dulbecco's Modified Eagle's Medium (DMEM) (Invitrogen, Paisley, Scotland) supplemented with 10% fetal bovine serum (Invitrogen, Paisley, Scotland), 300 mg/l L-glutamine (Invitrogen, Paisley, Scotland), 100 U/ml penicillin (Invitrogen, Paisley, Scotland) and 0.1 mg/ml streptomycin (Invitrogen, Paisley, Scotland), in a humidified incubator at 37°C in 5% CO_2 _atmosphere. Cells were grown to 80% confluence prior to experimental treatments.

### Chemicals

The following anti-neoplastic drugs were used in this study: LA-12, cisplatin (Pliva-Lachema a.s., Brno, Czech Republic), 17-AAG (Alexis Biochemicals, Lausen, Switzerland). LA-12 and cisplatin were dissolved in DMSO and diluted in cell medium to 0.5 mM stock solution and 17-AAG was dissolved in DMSO to 2 mM stock solution. Stock solutions of LA-12 and cisplatin were freshly prepared before use. The final concentration of DMSO in cell culture medium did not exceed 0.2%.

### SDS-PAGE and western blotting

SDS-PAGE and western blotting were performed as described previously [16]. The following antibodies were used: DO-1 mouse monoclonal antibody directed towards the epitope ^20^-SDLWKL-^25 ^within the p53 N-terminal region [32], CM-1 rabbit polyclonal antibody against human p53 protein (in house antibody), CHIP-11.1 mouse monoclonal antibody recognizing full length as well as truncated CHIP protein (in house antibody), Estrogen Receptor (clone SP1) rabbit monoclonal antibody (Lab Vision, Fremont CA, USA), CD1 mouse monoclonal antibody recognizing Cyclin D1 (in house antibody), EEV1-2.1 mouse monoclonal antibody against Hsp90 protein (in house antibody) and AC-40 monoclonal antibody recognizing actin (Sigma-Aldrich Inc. St. Louis, USA), which was used as a protein loading control. Final concentration of all antibodies used for immunodetection of proteins was 1 μg/ml in 5% milk containing 0.1% Tween 20 in PBS.

### Immunoprecipitation

Immunoprecipitation of conformationally different forms of p53 protein was performed using 1 μg of mouse monoclonal antibodies PAb1620 recognizing the wild-type conformation of human p53 protein at native form [33,34] and PAb240 recognizing the mutant conformations of p53 at native form [35], as described previously [33,35]. For immunoprecipitation of Hsp90 protein for platinum amount measurement by AAS, cells were lysed in 150 mM NaCl, 50 mM HEPES (pH 7.2) and 0.5% Tween 20 and sonicated. Protein concentrations were measured using Bradford's assay. 200 μl of cell lysate containing 400 μg of total protein was incubated overnight together with 2 μg of EEV2-1.1 mouse monoclonal antibody against Hsp90β protein (in house antibody) and AC88 mouse monoclonal antibody against Hsp90 protein (Stressgen Biotech Corp., Victoria, BC, Canada). 15 μl of G-protein sepharose beads (GE Healthcare, Wien, Austria) was used to precipitate the antibody. After three washes in lysis buffer, beads were resuspended in 200 μl of 150 mM NaCl, 50 mM HEPES (pH 7.2) and 0.5% Tween 20 and boiled for 10 min.

### Preparation of samples for AAS platinum amount measurement

Cells were incubated for the given time intervals with different doses of LA-12 and cisplatin. Control and treated cells were collected by centrifugation, washed three times with ice-cold PBS, lysed in 150 mM NaCl, 50 mM HEPES (pH 7.2) and 0.5% Tween 20 and sonicated. Protein concentrations were measured using Bradford's assay. 200 μl of cell lysate were used for platinum amount measurement by AAS.

### In vitro p53 DNA Binding Assay

Human wild-type p53 and human Hsp90β were purified and activity of p53 was quantified by EMSA as described previously [36]. Simplified, the assay is based on the ability of human recombinant Hsp90 to restore the specific DNA binding activity of human recombinant p53 at 37°C *in vitro*. A preincubation with Hsp90 inhibitors prior to p53 inactivation at 37°C was carried out as described for geldanamycin [21].

## List of abbreviations

AAS: Atomic absorption spectrometry; EMSA: Electrophoretic mobility shift assay; CHIP: Carboxy terminus of Hsp70-interacting protein; MDM2: Mouse double minute 2 homolog; Hsp90: Heat shock protein 90

## Competing interests

The authors declare that they have no competing interests.

## Authors' contributions

VK designed, performed and analysed platinum accumulation experiments, performed immunoprecipitation experiments and prepared the manuscript, RH designed and performed degradation experiments and prepared the manuscript, DW performed EMSA experiments, EM performed CHIP transfection experiments, VH and DS performed AAS analysis, PM and PS contributed to the experimental design of the study, BV contributed to the conception and design of the entire study and the final editing of the manuscript. All authors read and approved the manuscript.

## References

[B1] Ho YP Au-Yeung SC To KK Platinum-based anticancer agents: innovative design strategies and biological perspectives Med Res Rev 2003 23 633 655 10.1002/med.10038 12789689

[B2] Kozubik A Vaculova A Soucek K Vondracek J Turanek J Hofmanova J Novel Anticancer Platinum(IV) Complexes with Adamantylamine: Their Efficiency and Innovative Chemotherapy Strategies Modifying Lipid Metabolism Met Based Drugs 2008 2008 417 897 10.1155/2008/417897 PMC2291354 18414587

[B3] Choy H Park C Yao M Current status and future prospects for satraplatin, an oral platinum analogue Clin Cancer Res 2008 14 1633 1638 10.1158/1078-0432.CCR-07-2176 18347164

[B4] Fokkema E Groen HJ Bauer J Uges DR Weil C Smith IE Phase II study of oral platinum drug JM216 as first-line treatment in patients with small-cell lung cancer J Clin Oncol 1999 17 3822 3827 10577855 10.1200/JCO.1999.17.12.3822

[B5] Kelland LR An update on satraplatin: the first orally available platinum anticancer drug Expert Opin Investig Drugs 2000 9 1373 1382 10.1517/13543784.9.6.1373 11060749

[B6] Zak F Turanek J Kroutil A Sova P Mistr A Poulova A Mikolin P Zak Z Kasna A Zaluska D Platinum(IV) complex with adamantylamine as nonleaving amine group: synthesis, characterization, and in vitro antitumor activity against a panel of cisplatin-resistant cancer cell lines J Med Chem 2004 47 761 763 10.1021/jm030858+ 14736257

[B7] Turanek J Kasna A Zaluska D Neca J Kvardova V Knotigova P Horvath V SI L Kozubik A Sova P New platinum(IV) complex with adamantylamine ligand as a promising anti-cancer drug: comparison of in vitro cytotoxic potential towards A2780/cisR cisplatin-resistant cell line within homologous series of platinum(IV) complexes Anticancer Drugs 2004 15 537 543 10.1097/01.cad.0000127147.57796.e5 15166629

[B8] Horvath V Blanarova O Svihalkova-Sindlerova L Soucek K Hofmanova J Sova P Kroutil A Fedorocko P Kozubik A Platinum(IV) complex with adamantylamine overcomes intrinsic resistance to cisplatin in ovarian cancer cells Gynecol Oncol 2006 102 32 40 10.1016/j.ygyno.2005.11.016 16364413

[B9] Cermanova J Chladek J Soval P Kroutil A Semerad M Berankova Z Siroky P Surova I Single-dose pharmacokinetics of a novel oral platinum cytostatic drug ([OC-6-43]-bis[acetato][1-adamantylamine]amminedichloroplatinum [IV]) in pigs Methods Find Exp Clin Pharmacol 2004 26 679 685 10.1358/mf.2004.26.9.872565 15632953

[B10] Sova P Mistr A Kroutil A Zak F Pouckova P Zadinova M Preclinical anti-tumor activity of a new oral platinum(IV) drug LA-12 Anticancer Drugs 2005 16 653 657 10.1097/00001813-200507000-00010 15930894

[B11] Sova P Mistr A Kroutil A Zak F Pouckova P Zadinova M Comparative anti-tumor efficacy of two orally administered platinum(IV) drugs in nude mice bearing human tumor xenografts Anticancer Drugs 2006 17 201 206 10.1097/00001813-200602000-00012 16428939

[B12] Siddik ZH Cisplatin: mode of cytotoxic action and molecular basis of resistance Oncogene 2003 22 7265 7279 10.1038/sj.onc.1206933 14576837

[B13] Mellish KJ Barnard CF Murrer BA Kelland LR DNA-binding properties of novel cis- and trans platinum-based anticancer agents in 2 human ovarian carcinoma cell lines Int J Cancer 1995 62 717 723 10.1002/ijc.2910620612 7558420

[B14] Horvath V Soucek K Svihalkova-Sindlerova L Vondracek J Blanarova O Hofmanova J Sova P Kozubik A Different cell cycle modulation following treatment of human ovarian carcinoma cells with a new platinum(IV) complex vs cisplatin Invest New Drugs 2007 25 435 443 10.1007/s10637-007-9062-7 17520175

[B15] Roubalova E Kvardova V Hrstka R Borilova S Michalova E Dubska L Muller P Sova P Vojtesek B The effect of cellular environment and p53 status on the mode of action of the platinum derivative LA-12 Invest New Drugs 2010 28 4 445 53 10.1007/s10637-009-9270-4 19499188

[B16] Hrstka R Powell DJ Kvardova V Roubalova E Bourougaa K Candeias MM Sova P Zak F Fahraeus R Vojtesek B The novel platinum(IV) complex LA-12 induces p53 and p53/47 responses that differ from the related drug, cisplatin Anticancer Drugs 2008 19 369 379 10.1097/CAD.0b013e3282f7f500 18454047

[B17] Itoh H Ogura M Komatsuda A Wakui H Miura AB Tashima Y A novel chaperone-activity-reducing mechanism of the 90-kDa molecular chaperone HSP90 Biochem J 1999 343 Pt 3 697 703 10.1042/0264-6021:3430697 10527951 PMC1220604

[B18] Rosenhagen MC Soti C Schmidt U Wochnik GM Hartl FU Holsboer F Young JC Rein T The heat shock protein 90-targeting drug cisplatin selectively inhibits steroid receptor activation Mol Endocrinol 2003 17 1991 2001 10.1210/me.2003-0141 12869591

[B19] Shames DS Minna JD IP6K2 is a client for HSP90 and a target for cancer therapeutics development Proc Natl Acad Sci USA 2008 105 1389 1390 10.1073/pnas.0711993105 18230718 PMC2234151

[B20] Chakraborty A Koldobskiy MA Sixt KM Juluri KR Mustafa AK Snowman AM van Rossum DB Patterson RL Snyder SH HSP90 regulates cell survival via inositol hexakisphosphate kinase-2 Proc Natl Acad Sci USA 2008 105 1134 1139 10.1073/pnas.0711168105 18195352 PMC2234104

[B21] Walerych D Kudla G Gutkowska M Wawrzynow B Muller L King FW Helwak A Boros J Zylicz A Zylicz M Hsp90 chaperones wild-type p53 tumor suppressor protein J Biol Chem 2004 279 48836 48845 10.1074/jbc.M407601200 15358769

[B22] Muller P Hrstka R Coomber D Lane DP Vojtesek B Chaperone-dependent stabilization and degradation of p53 mutants Oncogene 2008 27 3371 3383 10.1038/sj.onc.1211010 18223694

[B23] Lukashchuk N Vousden KH Ubiquitination and degradation of mutant p53 Mol Cell Biol 2007 27 8284 8295 10.1128/MCB.00050-07 17908790 PMC2169174

[B24] Sova P Chladek J Zak F Mistr A Kroutil A Semerad M Slovak Z Pharmacokinetics and tissue distribution of platinum in rats following single and multiple oral doses of LA-12 [(OC-6-43)-bis(acetato)(1-adamantylamine)amminedichloroplatinum(IV)] Int J Pharm 2005 288 123 129 10.1016/j.ijpharm.2004.09.020 15607264

[B25] Csermely P Schnaider T Soti C Prohaszka Z Nardai G The 90-kDa molecular chaperone family: structure, function, and clinical applications. A comprehensive review Pharmacol Ther 1998 79 129 168 10.1016/S0163-7258(98)00013-8 9749880

[B26] Soti C Racz A Csermely P A Nucleotide-dependent molecular switch controls ATP binding at the C-terminal domain of Hsp90. N-terminal nucleotide binding unmasks a C-terminal binding pocket J Biol Chem 2002 277 7066 7075 10.1074/jbc.M105568200 11751878

[B27] Prodromou C Roe SM O'Brien R Ladbury JE Piper PW Pearl LH Identification and structural characterization of the ATP/ADP-binding site in the Hsp90 molecular chaperone Cell 1997 90 65 75 10.1016/S0092-8674(00)80314-1 9230303

[B28] Hawle P Siepmann M Harst A Siderius M Reusch HP Obermann WM The middle domain of Hsp90 acts as a discriminator between different types of client proteins Mol Cell Biol 2006 26 8385 8395 10.1128/MCB.02188-05 16982694 PMC1636778

[B29] Donnelly A Blagg BS Novobiocin and additional inhibitors of the Hsp90 C-terminal nucleotide-binding pocket Curr Med Chem 2008 15 2702 2717 10.2174/092986708786242895 18991631 PMC2729083

[B30] Yun BG Huang W Leach N Hartson SD Matts RL Novobiocin induces a distinct conformation of Hsp90 and alters Hsp90-cochaperone-client interactions Biochemistry 2004 43 8217 8229 10.1021/bi0497998 15209518

[B31] Fang L Ricketson D Getubig L Darimont B Unliganded and hormone-bound glucocorticoid receptors interact with distinct hydrophobic sites in the Hsp90 C-terminal domain Proc Natl Acad Sci USA 2006 103 18487 92 10.1073/pnas.0609163103 17130446 PMC1693689

[B32] Vojtesek B Bartek J Midgley CA Lane DP An immunochemical analysis of the human nuclear phosphoprotein p53. New monoclonal antibodies and epitope mapping using recombinant p53 J Immunol Methods 1992 151 237 244 10.1016/0022-1759(92)90122-A 1378473

[B33] Milner J Structures and functions of the tumor suppressor p53 Pathol Biol (Paris) 1997 45 797 803 9769943

[B34] Wang PL Sait F Winter G The 'wildtype' conformation of p53: epitope mapping using hybrid proteins Oncogene 2001 20 2318 2324 10.1038/sj.onc.1204316 11402327

[B35] Gannon JV Greaves R Iggo R Lane DP Activating mutations in p53 produce a common conformational effect. A monoclonal antibody specific for the mutant form Embo J 1990 9 1595 1602 1691710 10.1002/j.1460-2075.1990.tb08279.x PMC551855

[B36] Walerych D Olszewski MB Gutkowska M Helwak A Zylicz M Zylicz A Hsp70 molecular chaperones are required to support p53 tumor suppressor activity under stress conditions Oncogene 2009 28 4284 4294 10.1038/onc.2009.281 19749793

